# Mucosal vaccination with outer membrane vesicles derived from *Bordetella pertussis* reduces nasal bacterial colonization after experimental infection

**DOI:** 10.3389/fimmu.2024.1506638

**Published:** 2024-11-28

**Authors:** E. Rudi, E. Gaillard, D. Bottero, T. Ebensen, C. A. Guzman, Daniela Hozbor

**Affiliations:** ^1^ Laboratorio VacSal, Instituto de Biotecnología y Biología Molecular (IBBM), Facultad de Ciencias Exactas, Universidad Nacional de La Plata, CCT-CONICET La Plata, La Plata, Argentina; ^2^ Department of Vaccinology and Applied Microbiology, Helmholtz Centre for Infection Research, Braunschweig, Germany

**Keywords:** *Bordetella pertussis*, outer-membrane vesicles, mucosal, intranasal, IgA

## Abstract

**Introduction:**

We previously identified *Bordetella pertussis*-derived outer membrane vesicles (OMVs) as a promising immunogen for improving pertussis vaccines. In this study, we evaluated the efficacy of our vaccine prototype in immunization strategies aimed at reducing disease transmission by targeting colonization in the upper airways while maintaining protection against severe disease by reducing colonization in the lower respiratory tract.

**Methods:**

We assessed different mucosal administration strategies in a murine model, including homologous mucosal 2-dose prime-boost schedules and heterologous prime-boost strategies combining intramuscular (IM) systemic immunization with mucosal routes (intranasal, IN; or sublingual, SL). We utilized alum and c-di-AMP as adjuvants for the systemic and mucosal formulations of the OMV vaccine prototype, respectively. A homologous prime/boost IM immunization schedule and commercial vaccines were used for comparisons.

**Results:**

All tested heterologous schemes induced higher levels of specific IgG with significant avidity, as well as higher levels of IgG1 and IgG2c, compared to the corresponding homologous prime-boost 2-dose schemes via mucosal routes (OMV_IN-IN_ or OMV_SL-SL_). High IgA levels were observed post-*B. pertussis* challenge following OMV_IN-IN_ treatments and heterologous treatments where the second dose was administered via a mucosal route (prime-pull scheme). Furthermore, schemes involving the intranasal route, whether in a homologous or heterologous scheme, induced the highest levels of IL-17 and IFN-γ. Accordingly, these schemes showed superior efficacy against nasal colonization than the commercial vaccines. Homologous intranasal immunization exhibited the highest protective capacity against nasal colonization while maintaining an excellent level of protection in the lower respiratory tract. To further enhance protection against nasal colonization, we performed a comparative analysis of formulations containing either single or combined adjuvants, administered via homologous intranasal route. These assays revealed that the use of alum combined with c-di-AMP, did not enhance the immune protective capacity in comparison with that observed for the formulation containing c-di-AMP alone.

**Conclusions:**

All the experiments presented here demonstrate that the use of OMVs, regardless of the scheme applied (except for OMV_SL-SL_), significantly outperformed acellular pertussis (aP) vaccines, achieving a higher reduction in bacterial colonization in the upper respiratory tract (p<0.01).

## Introduction

Whooping cough is a respiratory infectious disease that affects individuals of any age but is more severe in young children who are unvaccinated or have incomplete vaccination schedules. In unimmunized infants, the disease can be lethal. Characterized by bouts of violent coughing, vomiting after coughing, cyanosis, and apneas, the disease is caused by the Gram-negative bacterium *B. pertussis* (1). Studies in animal models and even in humans have shown that a robust humoral immune response and IFN-γ, produced by Th1 cells, play a critical role in protection against the symptoms caused by primary infection with *B. pertussis* and also in adaptive immunity against reinfection (2–5). Recent studies using the non-human primate model (baboons) have shown that Th17 cells and IgA also play a role in protective immunity against this bacterium (6, 7).

Vaccination is the preferred prevention strategy for this contagious disease. Currently, two types of pertussis vaccines exist, with the first one developed consisting of non-replicating cells from the causative agent (wP). This formulation that induces potent Th1 and Th17 cell responses, as well as the establishment of a tissue-resident memory population (TRM) (8, 9) is effective in preventing pertussis in children up to 7 years old. Adverse effects associated with wP vaccines led to their non-recommendation for the adolescent and adult population and prompted the development of a new generation of more tolerable vaccines based on purified *B. pertussis* immunogens (acellular vaccines, aP) (10–13). The aP vaccines were introduced in the late 90s into routine immunization programs in many developed countries. In recent years, the incidence of pertussis has increased in several countries, including those with high vaccination coverage (14–17). Various explanations have been proposed for the resurgence of pertussis, including improvements in integrated disease surveillance, the prevalence of circulating *B. pertussis* strains that are more resistant—likely due to bacterial genomic and phenotypic changes driven by selective vaccine pressure—to immunity conferred by vaccination (especially that induced by acellular vaccines), and the more rapid waning of vaccine-induced immunity in the case of aP vaccines (18–24). The resurgence of the disease has prompted a short to medium-term response, leading to the incorporation of additional vaccine boosters in the adolescent and adult population, particularly in pregnant women (25, 26). The data collected so far indicate that pregnant women vaccination strategy is proving successful (27, 28).

However, significant challenges persist in vaccination strategies that demand closer examination. A major concern is that current vaccines, particularly acellular ones, fail to effectively reduce bacterial colonization in the upper respiratory tract (6, 29, 30). As a result, even vaccinated individuals can continue to transmit the disease. This is especially concerning, as vaccinated individuals who become infected often remain asymptomatic, unknowingly facilitating the silent transmission of the pathogen (6, 31, 32). Recently, it has been demonstrated that replacing alum with adjuvants promoting Th1 cells and/or TRM, including Toll-like receptor (TLR) agonists, can enhance the protective efficacy of experimental aP vaccines in mice (33–35). In particular, the combination of LP1569 (a ligand for TLR2) and the agonist for the intracellular receptor stimulating interferon genes, c-di-GMP, in an aP formulation induced significant numbers of respiratory IL-17-secreting CD4 TRM cells and prevented nasal colonization for at least 10 months after immunization (35). In preclinical assays, we have previously demonstrated that outer membrane vesicles (OMVs) derived from *B. pertussis* show promise for evaluation as a candidate for a third generation of pertussis vaccines. They have proven to be safe, induce a Th1/Th17/Th2 profile, generate TRM cells, and exhibit significant capability to reduce bacterial colonization in the lower airways, which is associated with severe disease (36–43). We detected this protective capacity with the OMV administered both systemically and mucosally (38, 43). Raeven et al. (44) conducted comparative studies between vaccination schemes using OMVs derived from another strain of *B. pertussis* administered subcutaneously and intranasally. The results obtained showed that intranasal administration of OMVs is an appropriate strategy to reduce transmission (bacterial colonization in upper tract) and severe disease (bacterial colonization in lower respiratory tract) when animals were exposed to bacterial suspensions containing 10^5^ CFU (44). In this study, aimed at further establishing OMVs as a viable immunization alternative. To this end, we assessed the impact of administering OMVs via different mucosal routes, sublingual and intranasal, in both homologous (same formulation and route for different doses) and heterologous (different formulations and routes for different doses) schemes on immunogenicity and protective capacity against bacterial colonization in the upper and lower respiratory tracts. Regarding the heterologous scheme, we incorporated a prime-pull strategy based on the rationale that intramuscular priming followed by an intranasal or sublingual booster would elicit robust systemic and mucosal immune responses. This approach may be particularly beneficial for booster vaccination in individuals who have already been immunized via the intramuscular route. We also assessed the protective efficacy of OMVs formulated with different adjuvants. For comparison purposes OMVs, aP and wP administered by intramuscular route were also evaluated.

## Materials and methods

### Mice

C57bl/6 mice (4 weeks old), obtained from the Faculty of Veterinary Sciences, La Plata, Argentina, were kept in ventilated cages and housed under standardized conditions with regulated daylight, humidity, and temperature. The animals received food and water ad libitum. The animal experiments were authorized by the Ethical Committee for Animal Experiments of the Faculty of Science at La Plata National University (approval number 004-06-15, 003-06-15 extended its validity until August 10, 2027).

### *B. pertussis* strain and growth conditions

*B. pertussis* Tohama phase I strain CIP 8132 was used throughout this study as the strain for challenge in the murine model of protection. Bacteria were grown in Bordet–Gengou (BG) agar supplemented with 10% (v/v) defibrinated sheep blood (BG-blood agar) for 72 h at 36.5°C. Isolated colonies were replated in the same medium for 24 h and then resuspended in phosphate-buffered saline (PBS: 123 mM NaCl, 22.2 mM Na_2_HPO_4_, 5.6 mM KH_2_PO_4_ in MilliQ^®^ nanopure water; pH 7.4). The optical density at 650 nm was measured and serial 10-fold dilutions plated onto BG-blood agar to determine the number of bacteria in the challenge inoculum.

### Isolation and characterization of outer membrane vesicles

OMVs were isolated and characterized as previously described (38, 45). Briefly, culture samples from the decelerating growth phase were centrifuged and the bacterial pellet obtained was resuspended in 20 mM Tris–HCl, 2 mM EDTA pH 8.5. The suspension was sonicated in cool water for 20 min. After two centrifugations at 10,000×g for 20 min at 4°C, the supernatant was pelleted at 100,000×g for 2 h at 4°C. This pellet was re-suspended in Tris buffer (20 mM pH 7.6). The samples obtained were negatively stained for electron microscope examination. Protein content was estimated by the Bradford method using bovine serum albumin as standard (46). The presence of the main immunogenic proteins in the OMVs was detected by immunoblot assays using specific antibodies as we previously described (not shown) (38, 40).

### Formulation of OMV-based vaccine

The characterized OMVs that range in size from approximately 50 to 200 nanometers in diameter were used to formulate the vaccine with tetanus (5 to 7 Lf/dose with a power greater than or equal to 2 UIA/ml serum) and diphtheria (1 to 3 Lf/dose with an output of 0.1 UIA/ml serum) toxoids as we previously described. OMVs formulated with alum (Alhydrogel 2%, CRODA), c-di-AMP (7.5 µg per dose) (Invivogen), or c-di-AMP (7.5 µg) combined with alum (Alhydrogel 2%) as adjuvants were evaluated throughout this study. To perform the experiments described below we verified that the OMV based vaccine prepared by us fulfilled the WHO criteria for safety in the weight-gain test (https://cdn.who.int/media/docs/default-source/biologicals/vaccine-standardization/pertussis/annex-6-whole-cell-pertussis.pdf?sfvrsn=f734b4_3&download=true). The safety of OMV-based vaccines was also confirmed by human whole-blood assays (47). This protocol evaluates the endotoxin activity of vaccines by measuring cytokine responses in a whole-blood setting. This approach allowed us to examine the safety and the tolerability of OMVs within a human-based ex vivo system, offering a relevant model to predict inflammatory potential and confirm the absence of excessive immune activation linked to OMV exposure.

### Immunization of mice

Groups of 4-8 female C57BL/6 mice were immunized with OMV-based vaccine formulated as previously described with 3-6 µg total protein per dose formulated with alum, c-di-AMP or a combination of both as adjuvant (described in the legends to the figures), or 1:10 human dose of aP vaccine BOOSTRIX^®^ [GlaxoSmithKline, with composition per human dose: pertussis toxoid (8 µg), pertactin (2.5 µg), filamentous hemagglutinin (8 µg), tetanus toxoid (20 IU), diphtheria toxoid (2 IU) and alum as adjuvant] or 1:10 human dose of whole cell vaccine (Serum Institute of India PVT LTD, composition per human dose is: diphtheria toxoid ≤ 25 Lf (≥ 30 IU), tetanus toxoid ≥ 5 Lf (≥ 40 IU), *B. pertussis* ≤ 16 OU (≥ 4 IU) adsorbed on aluminum phosphate ≤ 1.25 mg) or) using a 2-dose schedule. Two weeks after the last immunization blood samples were obtained. For protection assays mice were challenged with *B. pertussis* by intranasal inoculation (sublethal dose 1x10^7^- 5x10^7^ CFU 40μl^-1^) as is described below. Mice were sacrificed 1 week after challenge and bacterial counts were conducted following a previously established protocol (36, 37). Lungs and nose tissues were aseptically excised, homogenized in sterile PBS, serially diluted, and plated onto BG agar supplemented with defibrinated sheep blood to assess bacterial recovery at various time points post-infection. A minimum of three independent experiments were performed to ensure result robustness.

### Enzyme-linked immunosorbent assay

As we previously described (48), plates (Nunc A/S, Roskilde, Denmark) were coated with proteins from bacterial lysates at 3 µg/ml in 0.5 M carbonate buffer pH 9.5, by means of an overnight incubation at 4°C. Blocked plates with 3% milk in PBS (2 h 37°C) were incubated with serially diluted samples of mouse serum (1 h 37°C). Sera obtained after leaving the blood samples to clot for 1 h at 37°C followed by centrifuging for 10 min at 6,000xg. IgGs from individual serum or pooled sera bound to the plates were detected after a 2-h incubation with goat anti–mouse-IgG–linked horseradish peroxidase (1:8,000 Invitrogen, USA). For measuring IgG isotypes, detection of bound antibody was determined using HRP labeled subclass-specific anti-mouse IgG1 (1:3,000), IgG2c (1:2,000) (Invitrogen USA) or IgA (1/750) (Sigma Aldrich). As substrate 1.0 mg/ml o-phenylendiamine (OPD, Bio Basic Canada Inc) in 0.1 M citrate-phosphate buffer, pH 5.0 containing 0.1% hydrogen peroxide was used. Optical densities (ODs) were measured with Titertek Multiskan Model 340 microplate reader (ICN, USA) at 490 nm. From the experimental protocol performed in triplicate, one representative experiment is presented in the Results.

### Avidity assay

Avidity was measured by an ELISA elution assay as the overall strength of binding between antibody and antigen, using plates incubated for 10 min with increasing concentration of ammonium thiocyanate (NH_4_SCN) from 0 to 1 M. Antibody avidity was defined as the amount (percentage) of antibody retained for each increment of NH_4_SCN concentration.

### Ag- specific IL-17, IFN-γ and IL-5 production by spleen cells

After *B. pertussis* challenge, spleens from untreated and immunized mice were passed through a 40-mm cell strainer to obtain a single-cell suspension. Spleen cells (43) were seeded in 48 well culture plates in a final volume of 500 µl/well RPMI 1640 with 10% fetal bovine serum, containing 100 IU/ml penicillin and 100 µg/ml streptomycin. All cell samples were stimulated with OMV (2 µg/ml) or medium only. After 72 h of incubation (37°C and 5% CO_2_), IFN-γ, IL-5 and IL-17 concentrations were quantified in supernatants by ELISA (BD Biosciences, San Diego, USA), using conditions recommended by the manufacturer.

### Statistical analysis

The data were evaluated statistically by two-way or one-way analysis of variance (ANOVA) followed by Bonferroni for multiple comparisons (via the GraphPad Prism^®^ software). Differences were considered significant at a p <0.05.

## Results

### Immunogenicity of OMV based vaccine prototype administered via mucosal route through homologous or heterologous vaccination scheme

**Figure 1:**
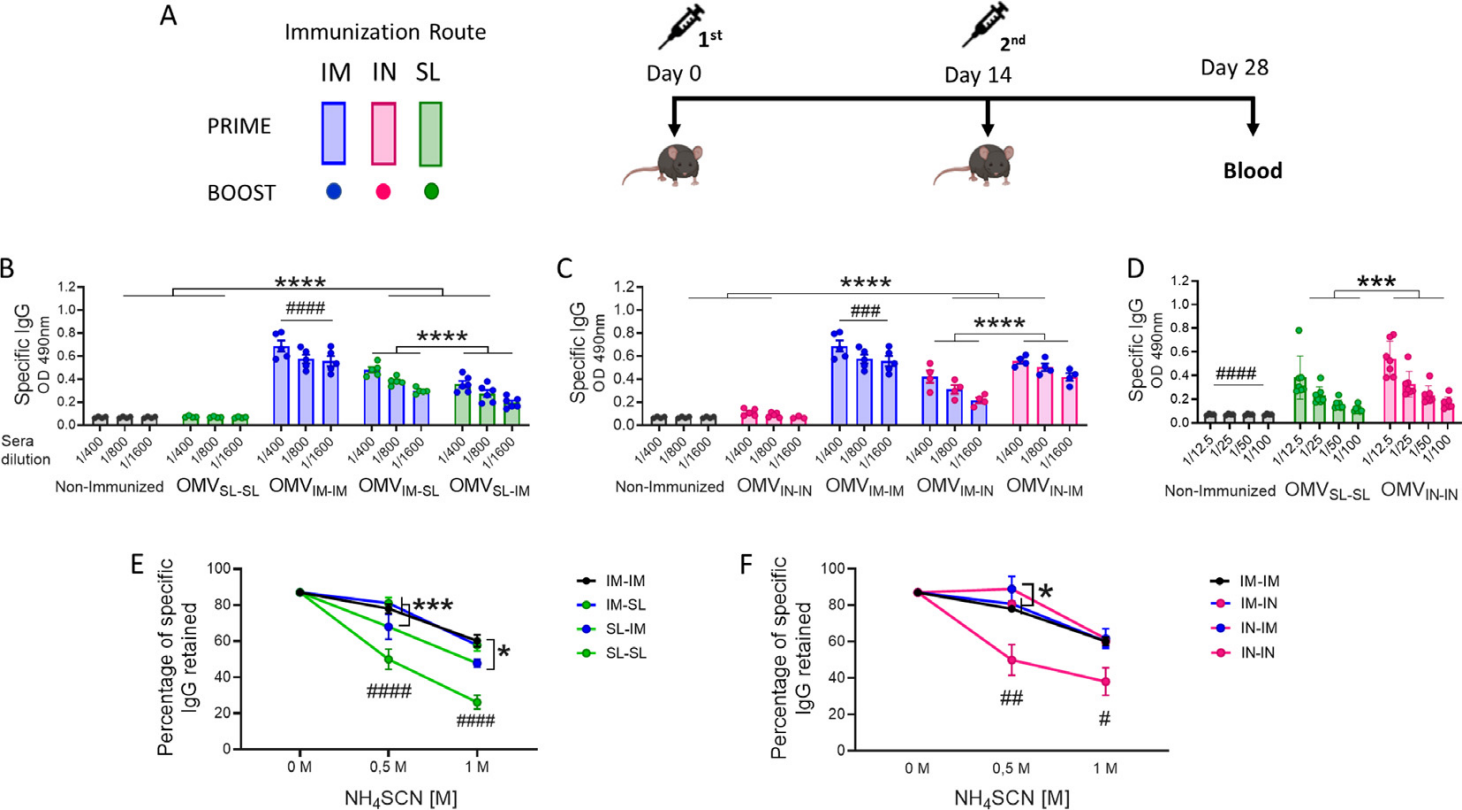
Characterization of the humoral immune response induced by OMVs administered through heterologous and homologous immunization schemes. C57BL/6 mice (n=7/group) were immunized on days 0 and 14, with a 2-dose scheme administered with a formulation containing OMVs using mucosal routes (sublingual OMV_SL_ or intranasal OMV_IN_) in homologous schemes (OMV_SL-SL_ or OMV_IN-IN_) or heterologous schemes (OMV_IM-SL_ OMV_SL-IM_, OMV_IM-IN_ or OMV_IN-IM_),. A homologous 2-dose scheme using the intramuscular route (OMV_IM-IM_) was also incorporated for comparative analysis. Different bars colors are used to discriminate the route used for the first dose and the colors of circles denote the route used for the booster dose **(A)**. Specific IgG levels induced by schedules that include SL administration **(B)**, IN administration **(C)** or homologous mucosal administration **(D)** were determined in sera collected on day 14 after the last dose (absorbance values at 490 nm for 2 sera dilutions). The avidity of IgG antibodies was also measured 14 days post the second dose and the results are presented as percentages of *B. pertussis*-specific IgG antibodies retained after exposure with 0.5 M and 1M of ammonium thiocyanate (NH_4_SCN) **(E, F)**. *p<0.05, ***p<0.001, ****p<0.0001 by two-way ANOVA using Bonferroni for multiple comparisons. The # symbol indicate significant differences between treatment and the other groups. #p<0.05, ##p<0.01, ###p<0.001, #### p <0.0001.

To assess the immunogenicity and then the ability of OMVs to reduce *B. pertussis* colonization in the lower and upper respiratory tract, groups of mice were immunized with a 2-dose scheme administered with a formulation containing OMVs using mucosal routes (sublingual OMV_SL_ or intranasal OMV_IN_) in homologous schemes (OMV_SL-SL_ or OMV_IN-IN_) or heterologous schemes (OMV_SL-IM_, OMV_IM-SL_, OMV_IN-IM_, OMV_IM-IN_). A homologous 2-dose scheme using the intramuscular route (OMV_IM-IM_) was also incorporated for comparative analysis. For the intramuscular systemic route, we employed a previously tested and proven effective formulation comprising OMVs at a final protein concentration of 3 μg and alum as adjuvant (38, 43). In the case of mucosal routes, formulations containing OMVs at a final protein concentration of 6 μg, supplemented with the mucosal adjuvant c-di-AMP, were utilized. We selected this adjuvant due to its promising results in mucosal immunization and its effectiveness in eliciting IFN-γ and IL-17 responses (49) —both essential for protection against *B. pertussis* (8, 29). The experimental setup and timelines of the experiment are shown in **Figure 1A**. High levels of serum *B. pertussis*-specific IgG were obtained with OMV_IM-IM_ and to a lesser extent in heterologous schemes, but not in homologous mucosal schemes or the non-immunized group (**Figures 1B, C**). Intranasal immunization elicited significantly higher *B. pertussis*-specific IgG antibody responses compared to sublingual immunization (p<0.001) (**Figures 1D**). Comparative avidity assays conducted with different concentrations of the chaotropic agent NH_4_SCN showed that among the homologous schemes, the OMV_IM-IM_ scheme had the *B. pertussis*-specific IgG with the highest avidity (p<0.01 when 0.5 M of NH_4_SCN was used (**Figure 1E, F**). Additionally, higher antibody affinity was observed in sera obtained from the heterologous schemes, with the lowest affinity detected in sera from mice immunized with OMV_SL-IM_ (**Figure 1E**).

**Figure 2:**
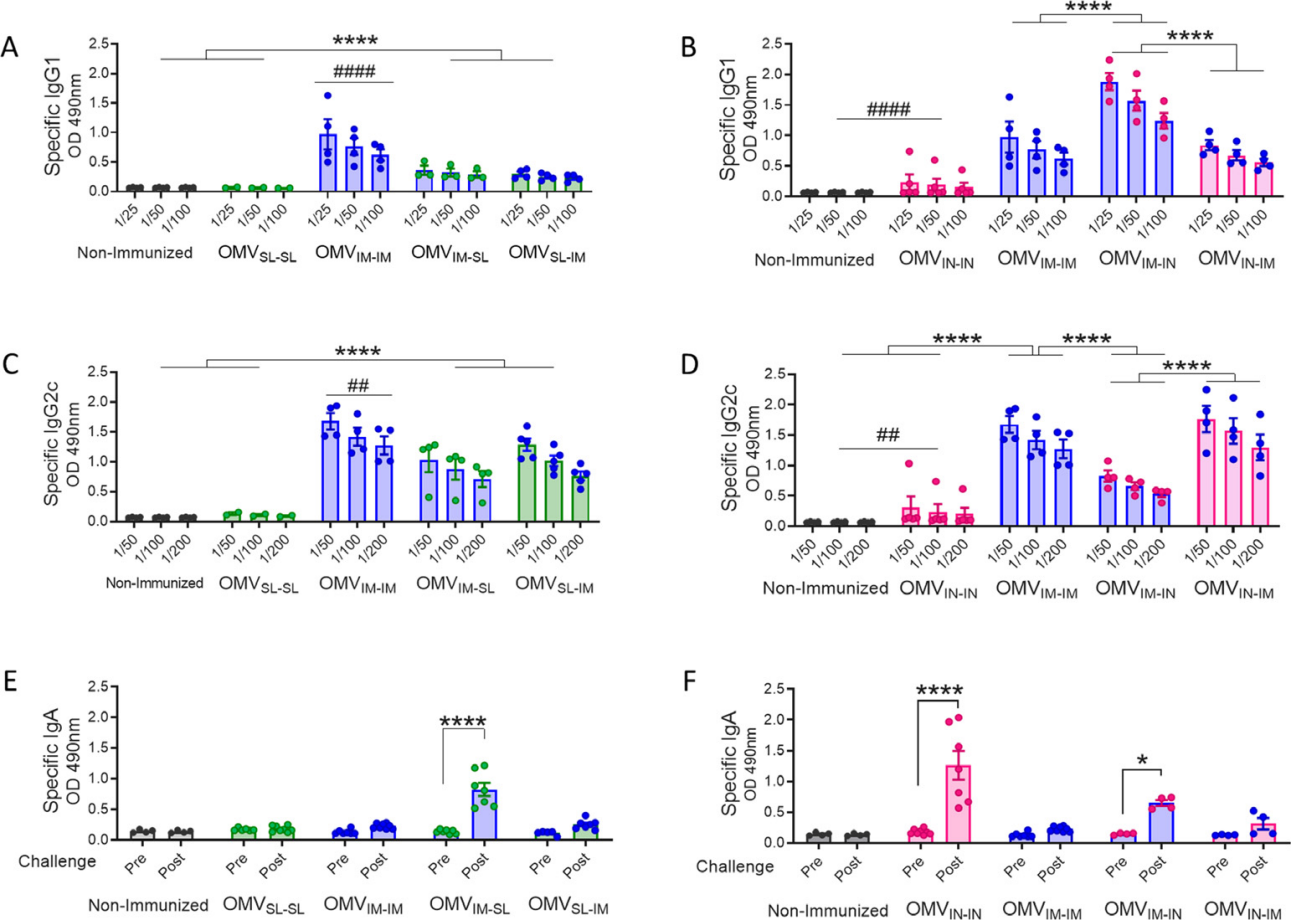
Specific *B. pertussis* IgG isotypes and IgA levels induced by OMVs administered through different heterologous and homologous immunization schemes. Groups of C57BL6 mice were immunized with a 2-dose scheme as described in panel A of **Figure 1**. A homologous 2-dose scheme using the intramuscular route was incorporated for comparative analysis (OMV_IM-IM_) and a group of non-immunized mice was employed as a control. Specific IgG1 **(A, B)** and IgG2a **(C, D)** levels were determined in sera of immunized mice two weeks after last dose (absorbance values at 490 nm for 3 sera dilutions). **(A, C)**: levels of IgG isotypes induced by schedules that include SL immunization. **(B, D)**: levels of IgG isotypes induced by schedules that include IN administration. IgA antibody responses in serum were evaluated before and after intranasal *B. pertussis* challenge (absorbance values at 490 nm) in the groups of immunized mice with schedules that include SL **(E)** and IN **(F)** administration. *p<0.05, ****p<0.0001 by two-way ANOVA using Bonferroni for multiple comparisons. The # symbol indicate significant differences between OMV_IM-IM_ and the other groups. # p<0.05, #### p <0.0001.

Regarding the levels of *B. pertussis*-specific IgG1 (**Figure 2A, B**), the highest levels were observed in the treatment group that received 2 doses of the OMV_IM_ formulation (**Figure 2A**), and in the heterologous scheme group OMV_IM-IN_ (p <0.0001). The levels detected for the prime-pull OMV_IM-IN_ scheme were significantly higher than those detected for OMV_IN-IM_ (p<0.0001, **Figure 2B**). Once again, the lowest levels of IgG1 were detected for the homologous schemes OMV_SL-SL_ or OMV_IN-IN_ (**Figure 2A, B**). No difference was detected in IgG1 levels between the mucosal homologous schemes.

When assessing IgG2c, the highest levels were found in the OMV_IN-IM_ and OMV_IM-IM_ treatments (**Figure 2C, D**). However, it is important to note that the heterologous schemes involving OMV_SL_ also induced significantly high levels, albeit lower than those observed in the former (**Figure 2C**). The IgG2c levels induced by OMV_IM-IN_ were higher than those induced by OMV_IN-IN_ (p<0.001). For mucosal homologous schemes and, as expected, the non-immunized control group, the levels of IgG2c isotypes were undetectable (**Figure 2C, D**). An interesting finding was that for all heterologous evaluated schemes except OMV_IM-IN_, the IgG2c/IgG1 ratios were higher than 1 (**Supplementary Table S1**), suggesting the induction of a Th1-directed immune response.

IgA antibody responses were only detected following intranasal *B. pertussis* challenge in mice treated with homologous intranasal immunization scheme and in those receiving the booster dose via mucosal route (prime-pull OMV_IM-IN_ and OMV_IM-SL_ heterologous treatments), whereas they were barely detectable in the other tested groups (**Figure 2E, F**).

**Figure 3:**
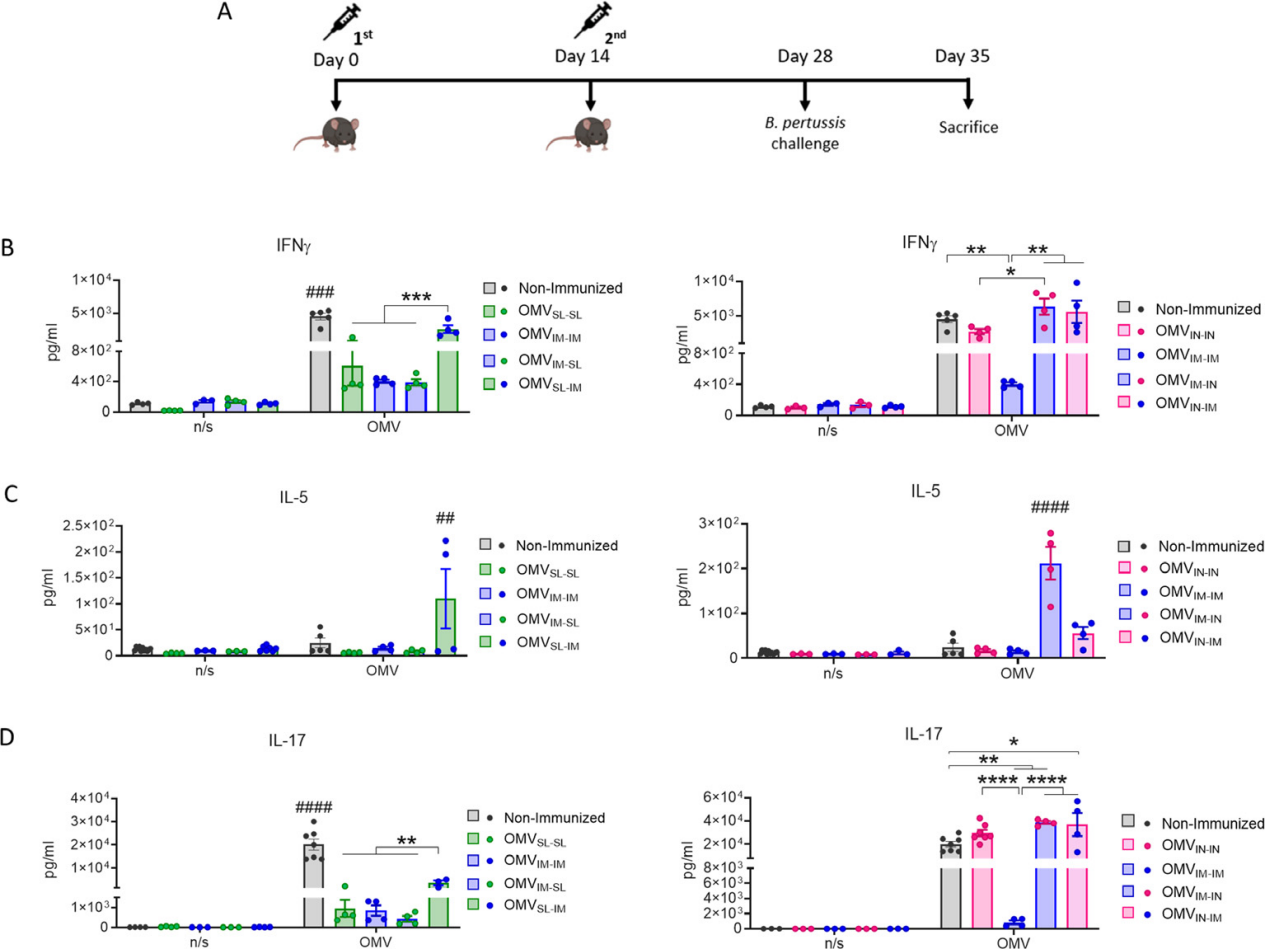
Characterization of cellular immune response induced by OMVs administered through heterologous and homologous immunization schemes. Mice were immunized on day 0 and 14, with a 2-dose scheme administered with a formulation containing OMVs using mucosal routes in homologous schemes or heterologous schemes, as described in **Figure 1**. Seven days after challenge with a sublethal dose (1x10^7^-5x10^7^ CFU/40 ul), mice were sacrificed, and their spleen cells were restimulated *ex-vivo* with 2μg/ml of OMV derived from *B. pertussis* or non-stimulated as controls (n/s). **(A)** Levels of secreted IFN-γ **(B),** IL-5 **(C)** and IL-7 **(D)** following splenocytes stimulation with medium or OMVs were determined by ELISA. Bars are means ± SEM of pg/ml. *p<0.05, **p<0.01, ***p<0.001, ****p<0.0001 by two-way ANOVA using Bonferroni for multiple comparisons. The # symbol indicate significant differences between the treatment and the other groups. ##p<0.01, ###p<0.001, #### p <0.0001.

IFN-γ (Th1 profile marker), IL-5 (Th2 profile marker) and IL-17 (Th17 profile marker) levels were also evaluated through splenocytes stimulation assays in mice challenged with a *B. pertussis* suspension (**Figure 3A**). The highest levels of OMV-specific IFN-γ and IL-17 were detected when OMV_IN_ was included, either in a homologous or heterologous routes of immunization, with no significant differences observed between the heterologous treatments (**Figure 3B, D**). It was also found that IL-17 levels in the heterologous scheme OMV_SL-IM_ were significantly higher than those induced by the other treatments containing any dose of OMV_SL_ (p<0.01).

The highest IL-5 levels were observed in heterologous schemes where the second dose was administered via IN route (**Figure 3C**).

These results demonstrate that the tested mucosal immunization schedules induced a robust specific humoral immune response when they were used in heterologous schemes showing higher levels of specific IgG in comparison with those detected for homologous mucosal schemes. It was also observed that the OMVs used in homologous or heterologous mucosal administration schemes induce a mixed Th1/Th17 profile, although more pronounced in schemes containing OMV_IN_ and in the heterologous scheme OMV_SL-IM_. IL-5 was also detected in some of the heterologous treatments here tested. High levels of IgA were detected for OMV_IN-IN_ and for prime-pull schemes OMV_IM-SL_ and OMV_IM-IN_.

### Protective capacity of OMV based vaccine prototype administered via mucosal route through homologous or heterologous vaccination schemes

**Figure 4:**
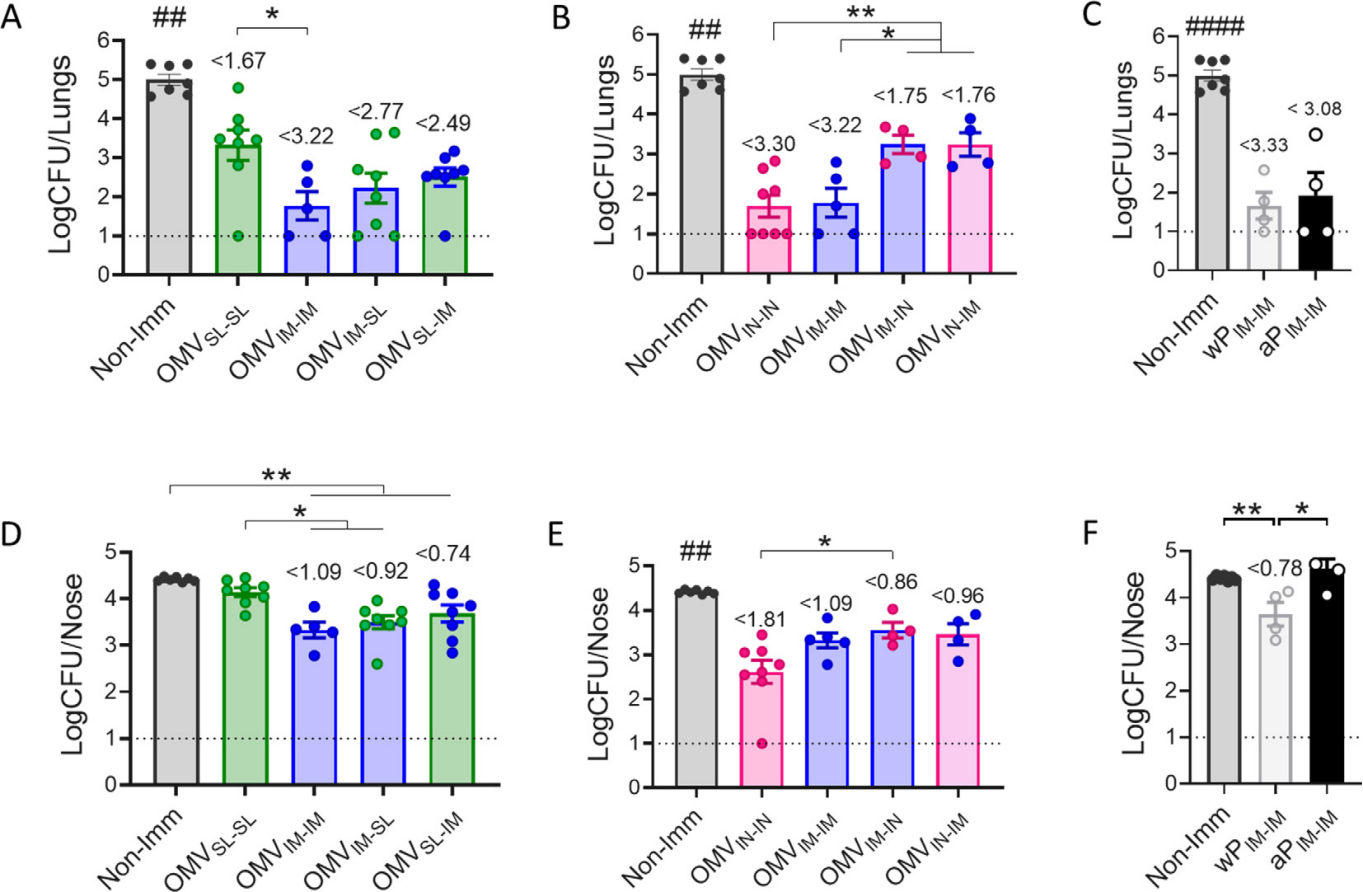
Effect of OMV mucosal homologous and heterologous immunization on protection against *B. pertussis* infection. C57BL/6 mice were immunized on days 0 and 14 with a 2-dose scheme OMV administered as described in panel A of **Figure 1**. Groups of animals immunized with commercial aP, or commercial wP vaccines or non-immunized were included as positive and negative protection control. Mice from all groups were challenged with a sublethal dose (1x10^7^ – 5 x10^7^/40 μl) *B. pertussis* Tohama phase I 14 days after the second dose followed by sacrifice 7 days after challenge. The number of bacteria recovered from mouse lungs **(A-C)** or nose **(D-F)** expressed as the log10 of CFUs per lungs or nose, is plotted on the ordinate, the different treatments here tested are indicated on the abscissa, with the data representing the means ± the SD. The dotted horizontal line indicates the lower limit of detection. The reduction detected in protection levels induced by different formulations in comparison with non-immunized animals is indicated at the top of the figures. *p<0.05, **p<0.01 by one way ANOVA using Bonferroni for multiple comparisons. The # symbol indicate significant differences between the treatment and the other tested groups. ## p<0.01, #### p <0.0001.

To assess the protective capacity exhibited by the different vaccination strategies tested here, groups of animals receiving 2-dose regimens were intranasally challenged with a suspension of *B. pertussis* at sublethal dose (1x10^7^ – 5x10^7^ CFU/40 μl) 14 days after receiving the last dose (**Figure 3A**). Seven days post-challenge mice, including those in the non-immunized group, were euthanized by cervical dislocation to evaluate bacterial colonization in the upper and lower respiratory tract. As expected, the non-immunized control group had a highest level of bacteria: 4.99x10^4^ CFU/lungs and 4.42x10^4^ CFU/nose (**Figure 4A, B**). Most immunized mice showed a significant reduction in the bacterial load in the lungs compared to non-immunized animals (p<0.0001), with the smallest reduction observed in the OMV_SL-SL_ immunized mice (reduction of 1.67 logs). For the heterologous treatments OMV_SL-IM_ and OMV_IM-SL_, significant reductions of 2.49 and 2.77 logs, respectively, were recorded. In the case of treatments that include an intranasal mucosal dose, reductions were 1.75 and 1.76 for mice immunized with OMV_IM-IN_ and OMV_IN-IM_ (**Figure 4B**). It is noteworthy that in the homologous schemes OMV_IN-IN_ and OMV_IM-IM_, reductions were at least 3.22 logs, similar to what was observed for commercial aP (reduction of 3.08 logs, **Figure 4C**) and wP (reduction of 3.33 logs, **Figure 4C**) vaccines administered intramuscularly.

Similar results were observed regarding the ability to reduce bacterial colonization in the upper respiratory tract (nose) (**Figure 4D, E**). The heterologous treatments showed a reduction of at least 0.74 logs (**Figure 4D, E**), similar to that detected for mice that received 2 doses of wP_IM_ (reduction of 0.78 logs) (**Figure 4F**). The heterologous schemes that induced the highest reduction were OMV_IM-SL_ and OMV _IN-IM_ with a reduction of at least 0.92 logs. The homologous schemes OMV_IN-IN_ (reduction of 1.81 logs) and OMV_IM-IM_ (reduction of 1.09 logs) but not OMV_SL-SL_, reduced bacterial colonization in the nose more effectively than the commercial aP vaccine applied in a 2-dose intramuscular scheme (no reduction) (p<0.001), (**Figure 4F**).

In summary, while homologous immunization with OMV_IN-IN_ showed the highest protective capacity in both upper and lower airways, heterologous schemes, particularly OMV_IM-SL_, exhibited adequate levels of protection surpassing the protection against nasal carriage conferred by the commercial vaccines.

### Immunogenicity and protective capacity of intranasal homologous scheme using formulation containing different adjuvants

With the aim of evaluating whether the combination of c-di-AMP and alum adjuvants can improve the efficacy of the best scheme identified thus far in this study (OMV_IN-IN_), we conducted comparative assays using formulations containing the adjuvant combination versus those with a single adjuvant. For our assays, a group of animals immunized with OMV formulated without any adjuvant was used as a control to assess the protective capacity of OMV alone.

The idea of testing the adjuvant combination, particularly with alum—though controversial—arises from recent findings showing the antibody production-enhancing effects of alum in nasal administration, as a consequence of increased IL-33 (50). It has been suggested that alum induces necroptosis of alveolar epithelial cells, followed by the release of IL-33, which enhances antigen-specific IgA antibody production by activating ILC2s and increasing the MHC class II level for antigen uptake by APCs (50).

**Figure 5:**
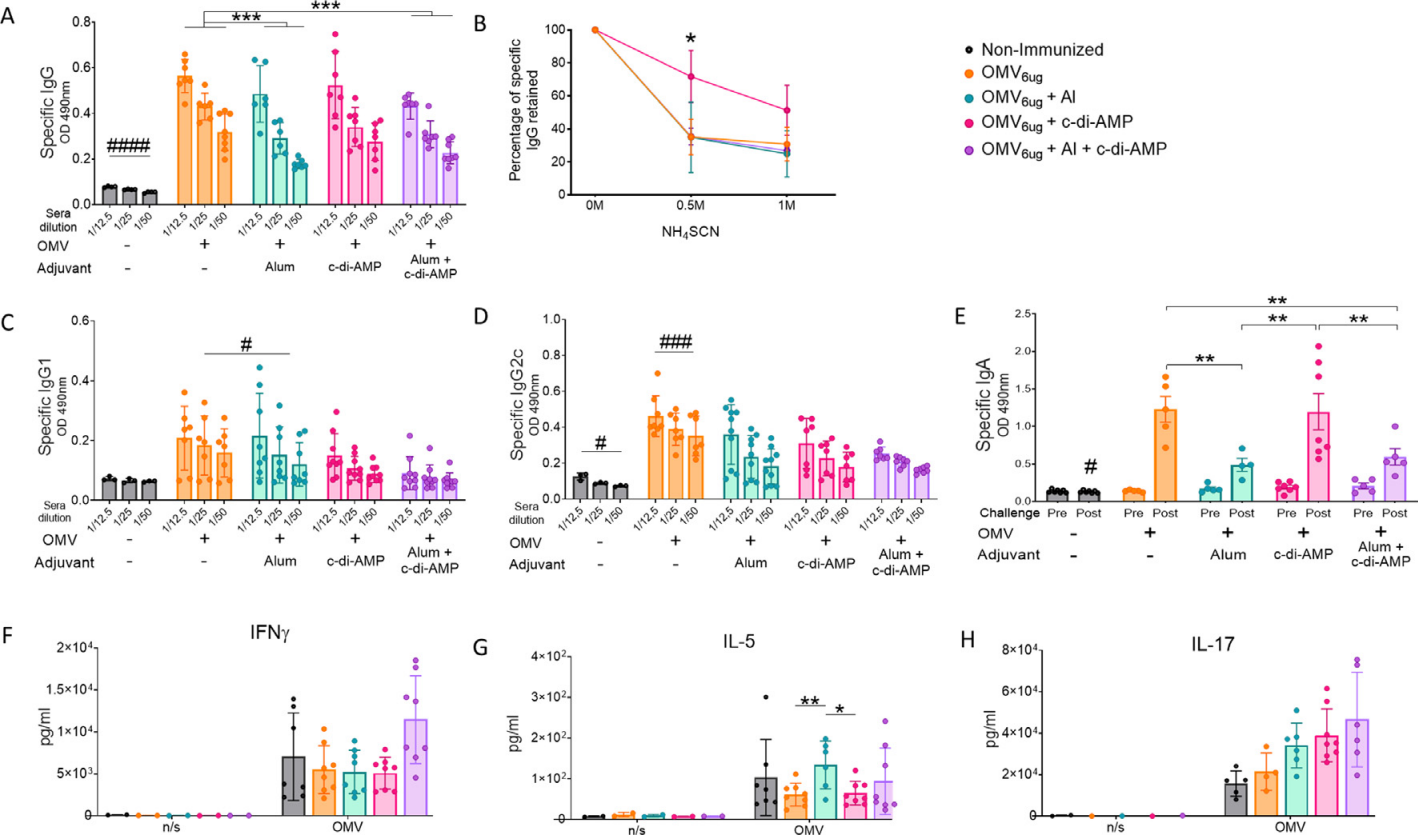
Characterization of immunogenicity and the protection capacity induce by OMVs administered by intranasal route using different adjuvants. Groups of C57BL6 mice were immunized with a 2-dose scheme administered IN with a formulation containing OMVs formulated with c-di AMP, Alum, or c-di AMP plus Alum. As a control, a group of non-immunized were employed. Specific IgG levels induced by different schedules were determined in sera collected on day 14 after the last dose (absorbance values at 490 nm) **(A)**. The avidity of IgG antibodies was also measured 14 days after the second dose and the results are presented as percentages of *B. pertussis*-specific IgG antibodies retained after exposure with 0.5 M and 1 M of ammonium thiocyanate (NH_4_SCN) **(B)**. Specific IgG1 **(C)** and IgG2c levels **(D)** were determined in sera of immunized mice two weeks after last dose (absorbance values at 490 nm). Mice from all groups were challenged with a sublethal dose (1x10^7^ – 5 x10^7^/40 μl) *B. pertussis* Tohama phase I 14 days after the second dose followed by sacrifice 7 days after challenge. IgA antibody responses in serum were detected before and after intranasal *B. pertussis* challenge (absorbance values at 490 nm) **(E)**. Levels of secreted IFN-γ **(F)**, IL-5 **(G)** and IL-7 **(H)** following splenocytes stimulation with medium or OMVs were determined by ELISA. Bars are means ± SEM of pg/ml. *p<0.05, **p<0.01, ***p<0.001 by two-way ANOVA using Bonferroni for multiple comparisons. The # symbol indicate significant differences between the treatment and the other groups. # p<0.05, ### p <0.001.

We again performed experiments involving 2-dose vaccination schemes. In these assays, we found that the addition of alum to formulations containing OMVs + c-di-AMP did not enhance IgG or IgG1 levels (**Figure 5A, C**). Furthermore, the highest antibody affinity was observed in the sera of mice that received two intranasal doses of OMVs formulated with c-di-AMP (**Figure 5B**). OMVs formulated without adjuvant induced higher IgG levels compared to those induced by the formulation with combined adjuvants. Additionally, OMVs without adjuvant or formulated with alum induced similar IgG1 levels, which were higher than those detected in the other treatments evaluated. Notably, the non-adjuvanted OMV group showed the highest increase in IgG2c levels (**Figure 5D**). In terms of IgA levels, both the group immunized with non-adjuvanted OMVs and the group treated with OMVs formulated with c-di-AMP exhibited the highest values (**Figure 5E**). No significant differences were observed in IFN-γ and IL-17 levels across all treatment groups (**Figure 5F, H**). A slight difference in IL-5 levels was detected, with the alum-adjuvanted OMV formulation inducing higher levels compared to both the c-di-AMP-adjuvanted and non-adjuvanted OMV formulations (**Figure 5G**).

**Figure 6:**
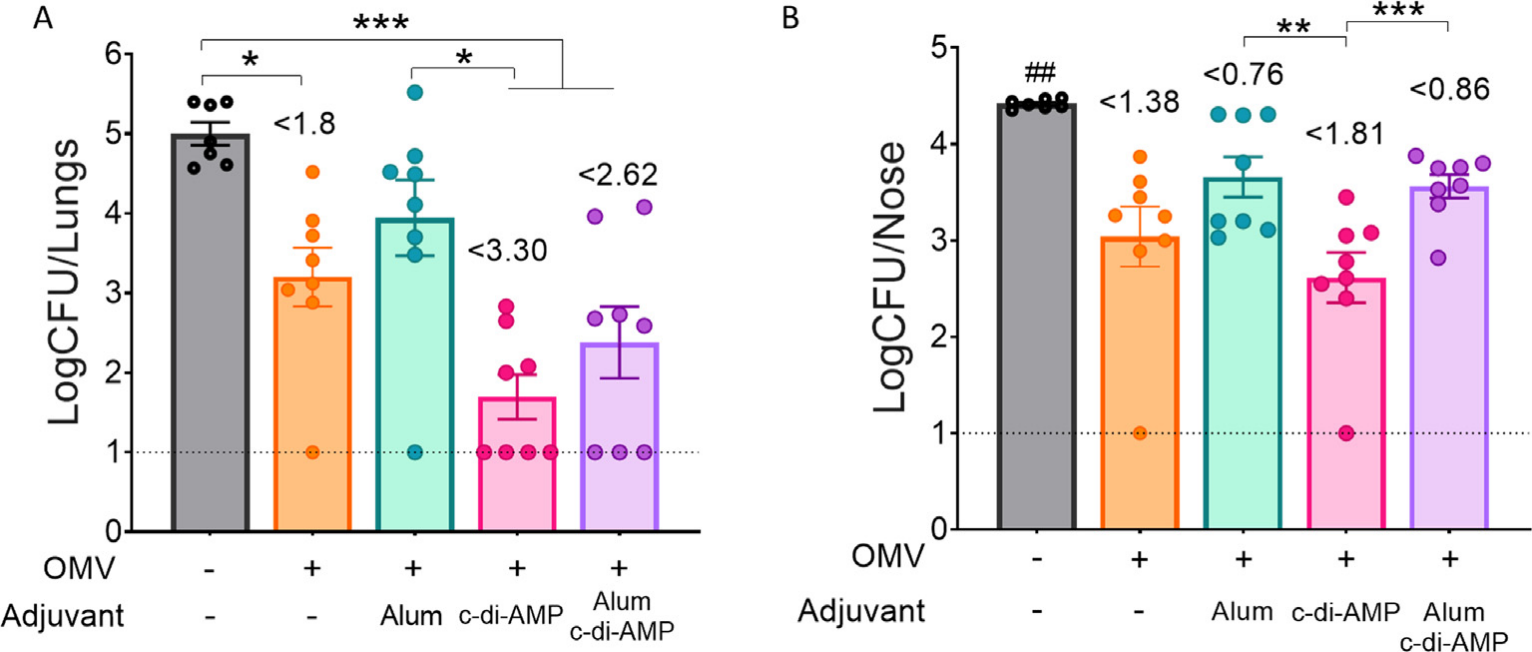
Effect of OMV intranasal homologous immunization on protection against *B. pertussis* infection. The number of bacteria recovered from mouse lungs (left panel **A**) or nose (right panel **B**) expressed as the log10 of CFUs per lungs or nose, is plotted on the ordinate, the different treatments here tested are indicated on the abscissa, with the data representing the means ± SEM. The reduction detected in protection levels induced by different formulations in comparison with non-immunized animals is indicated at the top of the figures. The dotted horizontal line indicates the lower limit of detection. p<0.05, **p<0.01, ***p<0.001, by one way ANOVA using Bonforroni for multiple comparisons. The # symbol indicate significant differences between the treatment and the other tested groups. ## p<0.01.

Regarding the efficacy in reducing bacterial colonization in the upper and lower respiratory tracts (**Figure 6**), the formulation containing the two adjuvants did not improve the protective capacity induced by OMVs+c-di-AMP formulation. In fact, the group of animals immunized with the c-di-AMP formulation showed the most substantial reduction. In the lower respiratory tract, bacterial colonization was reduced by 3.30 logs compared to non-immunized animals, whereas in the upper respiratory tract the reduction was 1.81 logs. The non-adjuvanted OMV formulation also induced a significant reduction in both the lower and upper respiratory tracts, with reductions of 1.8 logs and 1.38 logs, respectively. The lowest reduction in the upper respiratory tract was observed in the control group treated with OMVs formulated with alum (0.76 logs, **Figure 6**).

## Discussion

We have previously demonstrated that *B. pertussis* OMVs, which contain a diverse array of immunogens and PAMPs, represent a safe and effective vaccine prototype for preventing bacterial colonization in the lower respiratory tract (associated with severe disease) when administered either parenterally or intranasally (38, 42). Recently, we also demonstrated the adjuvant properties of OMVs when delivered through both systemic and mucosal routes (51). In this study, we evaluated the efficacy of *Bordetella pertussis*-derived outer membrane vesicles (OMVs) administered through mucosal immunization, specifically via sublingual or intranasal routes, to confer protective immunity not only in the lower respiratory tract but also in the upper respiratory tract, given its key role in disease transmission. We employed two-dose regimens involving either homologous (same route) or heterologous (different route) administration, including the prime-pull strategy, where the first dose is delivered systemically, and the second dose is administered via the mucosal route. In all cases involving mucosal immunization, a double dose of antigen (OMVs) was utilized compared to systemic administration, due to the partial loss of antigen crossing the mucosal barrier (50).

While many questions remain about sublingual immunization, such as identifying appropriate adjuvants and optimizing formulations to improve efficacy, there is a solid body of preclinical evidence demonstrating its safety and effectiveness in protecting against various respiratory pathogens, including influenza, RSV, and SARS-CoV-2 (52, 53). Regarding intranasal vaccination, the presence of abundant lymphatic tissue in the nasal cavity, which triggers both humoral (particularly the production of immunoglobulin A, accounting for more than 15% of total immunoglobulins) and cellular immune responses, has prompted numerous studies confirming its safety and efficacy (54).

Our findings show that OMVs formulated with c-di-AMP and administered via the sublingual route in a heterologous schedule, or intranasally in both homologous and heterologous schedules, significantly reduce bacterial colonization in both the upper and lower respiratory tracts. In heterologous schedules involving sublingual administration, colony counts in the lower respiratory tract were reduced by at least 309-fold, and by at least 5-fold in the upper respiratory tract. Interestingly, the heterologous prime-pull OMV_IM-SL_ schedule achieved the highest reductions, with a 588-fold decrease in the lungs and an 8-fold decrease in the nasal cavity compared to CFU counts in non-immunized mice (p< 0.01). Conversely, the least immunogenic and protective scheme was the homologous OMV_SL-SL_ schedule. The highest reductions in colonization in both the lungs and nose were observed in schedules that included at least one intranasal dose of OMVs formulated with c-di-AMP, with the OMV_IN-IN_ schedule yielding the highest reductions—1995-fold in the lungs and 64-fold in the nasal cavity—compared to non-immunized animals (p< 0.01). This result is consistent with the findings of Raeven et al. (44), who reported similar outcomes in a comparative study of OMV vaccination schemes. However, differences in colonization levels between their study and ours are likely attributable to variations in sampling methods (nasal lavages versus nasal tissue), the bacterial strains used to obtain OMVs, and the challenge doses administered.

Consistent with previously published data from baboon models, our study in mice further emphasizes the inherent limitations of acellular pertussis (aP) vaccines adjuvanted with alum in controlling bacterial colonization within the nasal cavity (6, 35). While intramuscular immunization with alum-adjuvanted aP vaccines effectively prevents lung infections, it does not significantly reduce nasal colonization by *B. pertussis*. Moreover, intranasal administration of the aP vaccine demonstrated poor protection against bacterial colonization in both the lungs and the nasal cavity, with colonization levels comparable to those of non-immunized animals (**Supplementary Figure S1**). As a result, the current formulation of this vaccine, regardless of whether it is delivered via the systemic or mucosal route, is unlikely to have a meaningful impact on horizontal transmission to susceptible hosts. In contrast, commercially available whole-cell pertussis (wP) vaccines, even when administered intramuscularly—as still practiced in several countries, including Argentina—demonstrated a substantial reduction in lung colonization (approximately 2000-fold) and nasal colonization (6-fold) compared to non-immunized controls. Likewise, OMV-based formulations, when administered systemically in a homologous two-dose scheme, significantly reduced bacterial colonization in both the lungs and nasal cavity, achieving over a 1600-fold reduction in the lungs and a 12-fold reduction in the nasal cavity relative to non-immunized animals.

Our findings underscore the superior efficacy of OMV-based formulations, particularly the OMV_IN-IN_ schedule containing OMVs formulated with c-di-AMP, when compared to currently available vaccines. Even though the formulation of OMVs with combined adjuvants (c-di-AMP plus alum) did not enhance the overall efficacy compared to OMVs with c-di-AMP alone, it still demonstrated substantial reductions in colonization in both the lower and upper respiratory tracts. Importantly, this dual-adjuvant formulation showed a clear advantage over the acellular pertussis vaccine, especially in reducing colonization in the upper respiratory tract. Notably, even OMVs without any adjuvant significantly reduced nasal colonization, achieving a 24-fold decrease relative to non-immunized animals (p < 0.01).

Another important finding of our study is that prime-pull schemes, while less effective than the homologous OMV_IN-IN_ schedule, demonstrated superior results in terms of the reduction of nasal colonization compared to the currently used commercial vaccines, specifically both wP and aP. In fact, these schemes, which delivered the second dose via the mucosal route, similarly to the homologous OMV_IN-IN_ schedule, induced elevated IgA levels post-challenge. Furthermore, schedules incorporating an intranasal dose (either homologous or heterologous) elicited high levels of IL-17, a cytokine described as crucial for protection in the upper respiratory tract. Previous studies from our team showed the intrinsic capacity of intranasal vaccination to promote per se strong IL-17 responses (55). In this regard, Solans et al. (56) highlighted the importance of IL-17 and IgA in mediating protection against *B. pertussis* nasopharyngeal colonization, emphasizing the differential mechanisms of protection in the upper (IgA and IL-17) and lower respiratory tract, where complete bacterial clearance primarily depends on cellular immunity mediated by T-helper type 1 (Th1) and Th17 cells (4).

In conclusion, OMV-containing formulations represent a promising prototype that should be considered for further evaluation as a third-generation vaccine against pertussis. When formulated with c-di-AMP and administered via the intranasal route, whether in homologous or heterologous schedules, these formulations effectively induce humoral, IL-17, and IFN-γ responses, offering protection against *B. pertussis* colonization in the lungs, but also in the nasal cavity, at levels superior to those observed with currently available commercial vaccines.

## Author’s note

Since the beginning of 2024, the government has decided to defund science, and as a result, the money from our grants has not been transferred to the funding units of our institutions so far.

## Data Availability

The datasets presented in this study can be found in online repositories. The names of the repository/repositories and accession number(s) can be found in the article/**Supplementary Material**.

## Supplementary material

Supplementary Figure 1 Effect of aP immunization on protection against *B. pertussis* infection. C57BL/6 mice were immunized on days 0 and 14, with a 2-dose scheme aP administered by intramuscular or intranasal route. Mice from all groups were challenged with a sublethal dose (1x10^7^ – 5 x10^7^/40 μl) *B. pertussis* Tohama phase I 14 days after the second dose followed by sacrifice 7 days after challenge. The number of bacteria recovered from mouse lungs **(A)** or nose **(B)** expressed as the log10 of CFUs per lungs or nose, is plotted on the ordinate, the different treatments here tested are indicated on the abscissa, with the data representing the means ± SEM. The dotted horizontal line indicates the lower limit of detection. The reduction detected in protection levels induced by different formulations in comparison with non-immunized animals is indicated at the top of the figures. ****p<0.0001 by one way ANOVA using Bonferroni for multiple comparisons.
