# Supplementary figures and images for: Mucosal vaccination with outer membrane vesicles derived from Bordetella pertussis reduces nasal bacterial colonization after experimental infection
Source: Front Immunol. 2024 Nov 28;15:1506638. doi: 10.3389/fimmu.2024.1506638 (PMC11635837; doi:10.3389/fimmu.2024.1506638)

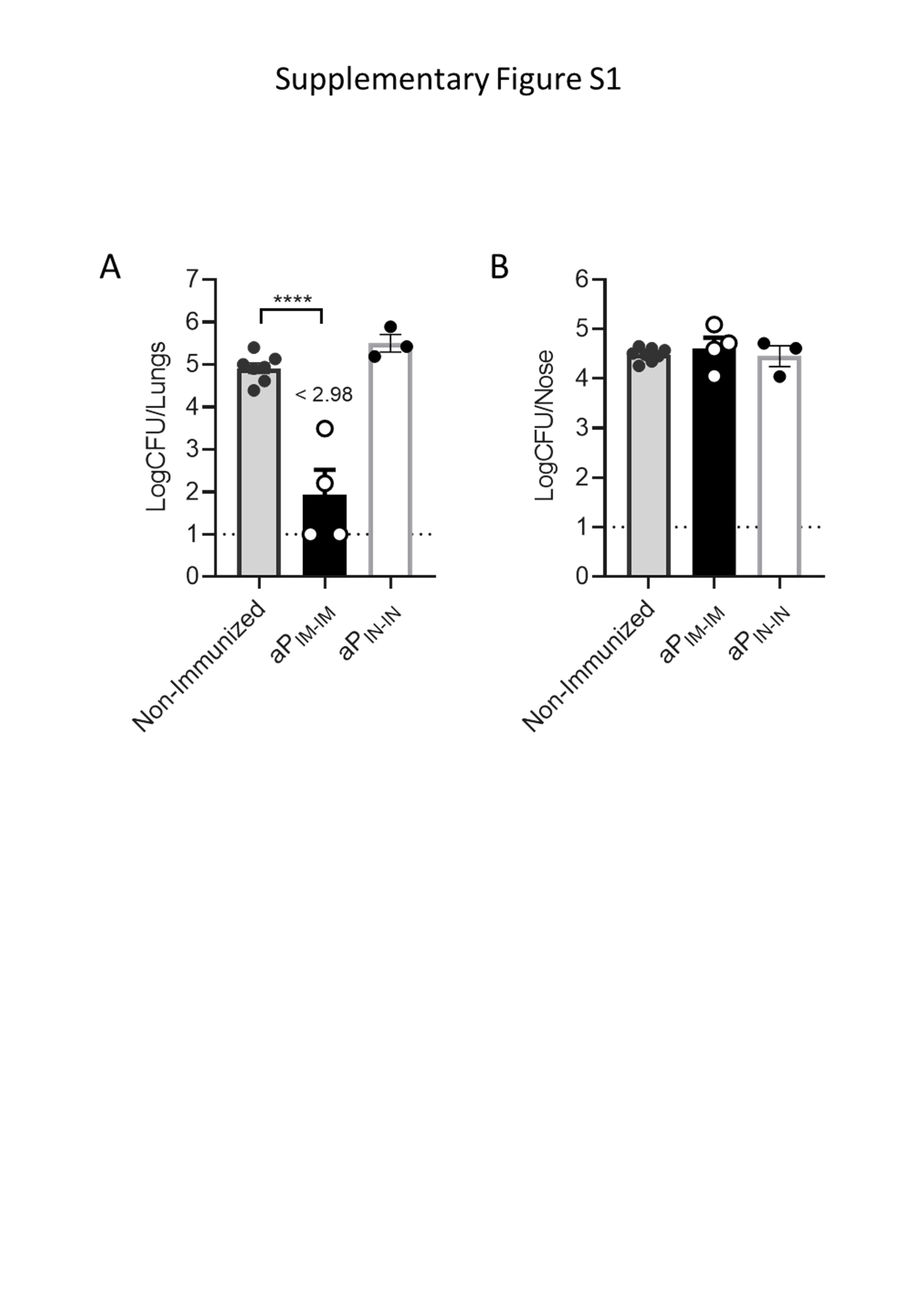

Supplement: Supplementary Figure 1 — Effect of aP immunization on protection against B. pertussis infection. C57BL/6 mice were immunized on days 0 and 14, with a 2-dose scheme aP administered by intramuscular or intranasal route. Mice from all groups were challenged with a sublethal dose (1x10^7^ – 5 x107/40 μl) B. pertussis Tohama phase I 14 days after the second dose followed by sacrifice 7 days after challenge. The number of bacteria recovered from mouse lungs (A) or nose (B) expressed as the log10 of CFUs per lungs or nose, is plotted on the ordinate, the different treatments here tested are indicated on the abscissa, with the data representing the means ± SEM. The dotted horizontal line indicates the lower limit of detection. The reduction detected in protection levels induced by different formulations in comparison with non-immunized animals is indicated at the top of the figures. ****p<0.0001 by one way ANOVA using Bonferroni for multiple comparisons. [file Image1.tif]

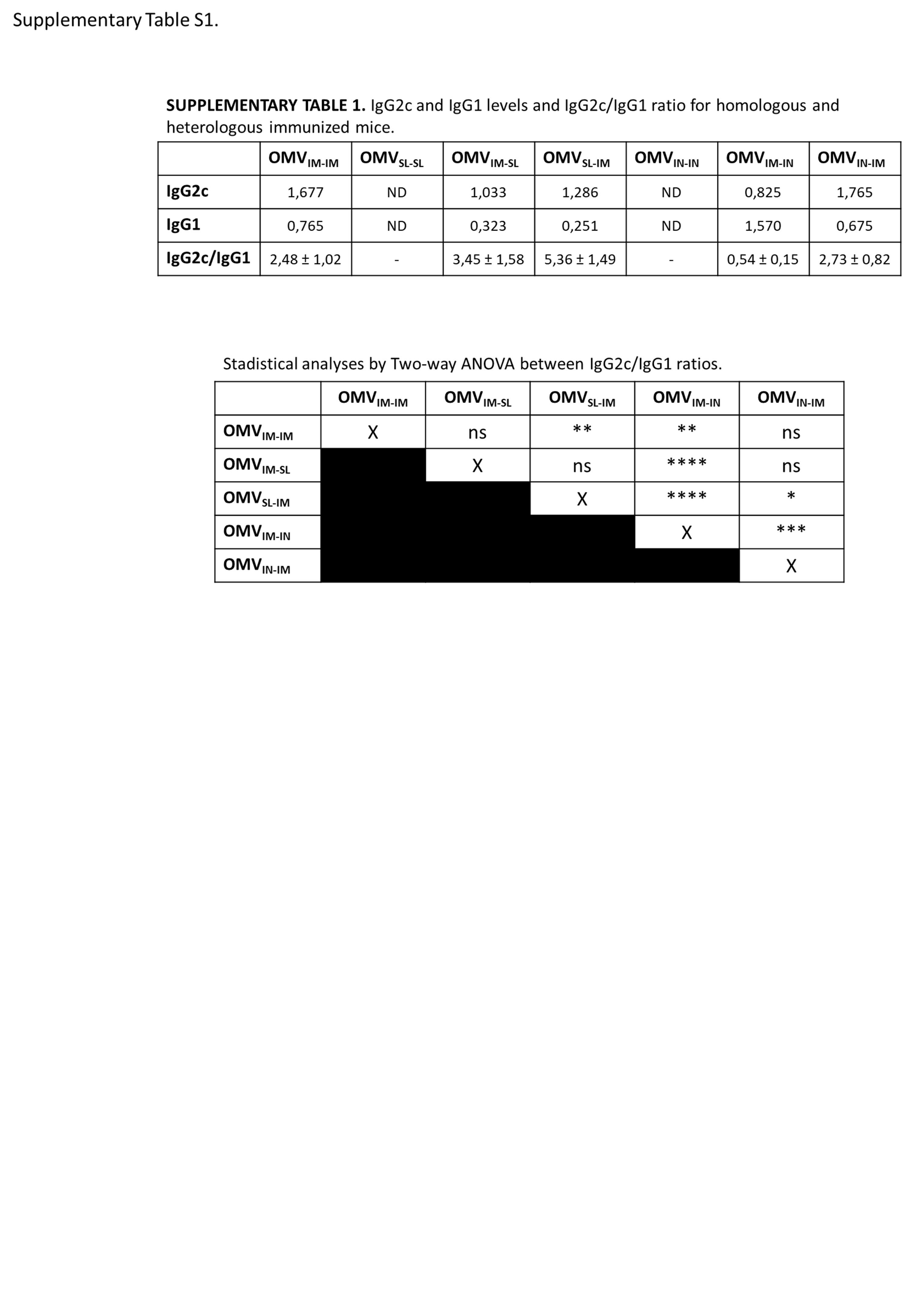

Supplement: Supplementary file 2 [file Image2.tif]
